# Alien Crabs as Potential Hosts of Pathogens Impacting Marine Megafauna’s Health and Conservation

**DOI:** 10.3390/pathogens12091177

**Published:** 2023-09-19

**Authors:** Giovanni Di Guardo

**Affiliations:** General Pathology and Veterinary Pathophysiology, Veterinary Medical Faculty, University of Teramo, Località Piano d’Accio, 64100 Teramo, Italy; gdiguardo@unite.it

Climate change, with a special emphasis on global warming, is believed to be a key driver of the accelerated rate of alien species expansion into the Mediterranean Sea basin and, more generally, into all marine and oceanic ecosystems [1]. Indeed, the last 8 years (2015–2022) have been the hottest recorded in the last 140 years [2]. 

To provide a numerical representation of the magnitude of such an alarming phenomenon, 70 new species were added in the period of 2017 to 2019 to the list of those inhabiting the Mediterranean Sea [1], including blue swimming crabs, *Callinectes sapidus*, which commonly occur in the central Adriatic Sea [3], and *Portunus segnis*, which has recently been reported in the same geographical area [4].

The colonization of marine and oceanic ecosystems different to those where a given species, either vertebrate or invertebrate, is endemic and is “traditionally” found has several implications, among which the subsequently arising alterations of sea food webs and food chains are of special concern, together with an increased risk of the introduction of “new” and/or emerging infectious agents, potentially carried by alien species into the marine and ocean environments hosting them. Furthermore, since extreme weather events, like the exceedingly frequent floods, represent the “alter ego” of global warming within the climate change *scenario* characterizing the present “Anthropocene Epoch”, adequate attention should be also paid to the land-to-sea transfer of a large number of oro-faecally transmitted viral, bacterial, fungal and protozoan pathogens, impacting the health and conservation *status* of marine vertebrate organisms, with special emphasis on inshore or coastal species. This is the case, for instance, for *Toxoplasma gondii*, a zoonotic and protozoan agent of global concern to free-living cetaceans, whose health and conservation *status* appear to be increasingly threatened by human activities as well as by several anthropogenic and non-anthropogenic factors, often acting synergistically with each other [5].

It is noteworthy that three new *Flavivirus* genus members—RNA viruses classified into the *Flaviviridae* family—have been recently discovered in crustaceans (*Gammarus chevreuxi flavivirus*, *Gammarus pulex flavivirus* and *Crangon crangon flavivirus*), with *Wenzhou shark flavivirus* (WSFV) variants having been additionally documented in several gazami crab (*Portunus trituberculatus*) populations. These findings strongly support the assumption that WSFV moves horizontally between sharks and gazami crabs in ocean ecosystems [6]. 

*P. trituberculatus*, the most extensively fished crab worldwide and a crustacean model that has been investigated in depth, is widely distributed in the Indian and West Pacific oceans, with a ubiquitous presence in southeast/east Asian countries as well as in northern and eastern Australian coastal waters [7].

While the ability of flaviviruses to infect a range of vertebrate species has been either gained or lost throughout time [8], the aforementioned study has provided clear-cut evidence of an intriguing vertebrate–invertebrate host association in flaviviral evolution, although it was not possible to establish whether WSFV jumped from sharks to *P. trituberculatus* or *viceversa* [6]. In this respect, it has already been shown that other RNA viral agents like rhabdoviruses have occasionally moved between distantly related species, subsequently spreading into closely related hosts [9]. While it is still unknown whether any pathological changes are caused by WSFV in susceptible host species, it should be also emphasized that viral genomes and/or antigens are abundantly found within all body tissues of infected sharks [8].

Several shark species are known to populate the Adriatic and the Mediterranean Sea more generally, with the health and conservation *status* of a number of them being increasingly threatened by both anthropogenic and non-anthropogenic factors, which largely justifies their inclusion on the "Red List" of the "International Union for the Conservation of Nature" (IUCN) [10].

Based upon the documented occurrences in the central Adriatic Sea of *C. sapidus* and *P. segnis* [3,4], both of which are closely related to *P. trituberculatus*, it would be interesting to investigate whether WSFV or other “cousin” flaviviruses circulate between these two alien crustaceans, as well as between them and one or more shark species and populations residing in the same geographic area.

Within this framework, it should be additionally emphasized that the *West Nile virus* (WNV), a relevant zoonotic, arthropod-borne flaviviral pathogen infecting humans and a huge number of mammalian, avian, reptilian and amphibian species [11], has been also deemed able to infect aquatic mammals, with WNV-infected common seals (*Phoca vitulina*) and killer whales (*Orcinus orca*) developing fatal polioencephalomyelitis [12] and non-suppurative encephalitis [13], respectively, in a similar fashion to WNV-infected horses [14] and human patients [15].

As a concluding remark, while the circulation of flaviviruses between marine and oceanic vertebrates and invertebrates, along with their potential susceptibility to flaviviral infections, undoubtedly represents a challenging and intriguing issue warranting ad hoc research efforts, I believe that a "One Health"-based approach should be the necessary prerequisite to adequately investigate these complex host–pathogen interactions and coevolutionary dynamics, since human health, animal health and environmental health are tightly and mutually linked to one another.
